# Overexpression of efflux pump genes is one of the mechanisms causing drug resistance in *Mycobacterium tuberculosis*

**DOI:** 10.1128/spectrum.02510-23

**Published:** 2023-12-04

**Authors:** Ying Long, Bin Wang, Tiancheng Xie, Ruixin Luo, Jing Tang, Jianping Deng, Chuan Wang

**Affiliations:** 1 West China School of Public Health and West China Fourth Hospital, Sichuan University, Chengdu, China; 2 Zigong Center for Disease Control and Prevention, Zigong, China; Yangzhou University, Yangzhou, Jiangsu, China

**Keywords:** *Mycobacterium tuberculosis*, MIRU-VNTR, mutation, efflux pump gene, verapamil, drug resistance mechanism

## Abstract

Currently, drug resistance is a formidable obstacle to tuberculosisprevention globally. A deep understanding of the drug-resistance mechanism of *Mycobacterium tuberculosis* is helpful to deal with this problem. In this study, 46 clinical isolates of *Mycobacterium tuberculosis* from Zigong, Sichuan, China, including 5 sensitive isolates, 5 rifampicin (RIF) mono-resistant isolates, 18 isoniazid mono-resistant isolates, and 18 multi-drug-resistant isolates, were selected. We evaluated the impact of drug induction on the relative expression levels of 10 putative efflux pump genes, namely, *Rv3065*, *Rv2836c*, *efpA*, *Rv1410c*, *Rv1250*, *Rv0876c*, *Rv1819c*, *Rv0933*, *Rv1217c*, and *Rv1218c*. Meanwhile, we assessed the effect of verapamil, an efflux pump inhibitor, on the drug resistance of the isolates. The results showed that 100% (5/5) of the RIF mono-resistant isolates, 44.4% (8 of 18) of the isoniazid mono-resistant isolates, and 88.9% (16 of 8) of the multi-drug-resistant isolates overexpressed anyone of the 10 efflux pump genes; however, none of the 10 efflux pump genes were overexpressed on the sensitive isolates. Among drug-resistant strains, *Rv1250* (51.2%) and *Rv0933* (53.7%) were the genes with the highest frequency of overexpression. Only the rifampicin mono-resistant isolates showed a significant increase in the number of overexpressed efflux pump genes after drug induction, while the change trends of the numbers of overexpressed efflux pump genes in isoniazid mono-resistant isolates and multi-drug-resistant isolates after drug induction were not definite. Verapamil did not have a significant effect on the minimum inhibitory concentrations (MICs) of isoniazid mono-resistant isolates but reduced the MICs of all sensitive isolates and some multi-drug-resistant isolates. We also investigated the correlation between polymorphic loci of mycobacterial interspersed repetitive unit-variable number tandem repeat (MIRU-VNTR) typing, mutations in drug resistance-associated genes, and overexpression of the efflux pump genes. The results indicated that there was no significant association between the two most polymorphic loci, MIRU26 and MIRU31, and the incidence of overexpression of the efflux pump genes. However, isolates with mutations in drug resistance-associated genes exhibited a higher rate of overexpression of the efflux pump genes compared to those without mutations. In conclusion, we found that drug-resistant isolates had a higher background expression level of the efflux pump genes compared to sensitive isolates. This suggests that the overexpression of the efflux pump genes could be one of the mechanisms causing drug resistance in *Mycobacterium tuberculosis*. We also found that verapamil was able to reduce MICs in some drug-resistant isolates, indicating that inhibition of the efflux pump could be used as an adjuvant treatment option for drug-resistant tuberculosis patients.

**IMPORTANCE:**

Gene mutations cannot explain all drug resistance of *Mycobacterium tuberculosis*, and the overexpression of efflux pump genes is considered another important cause of drug resistance. A total of 46 clinical isolates were included in this study to analyze the overexpression of efflux pump genes in different resistant types of strains. The results showed that overexpression of efflux pump genes did not occur in sensitive strains. There was no significant trend in the overexpression of efflux pump genes before and after one-half of MIC drug induction. By adding the efflux pump inhibitor verapamil, we can observe the decrease of MIC of some drug-resistant strains. At the same time, this study ensured the reliability of calculating the relative expression level of efflux pump genes by screening reference genes and using two reference genes for the normalization of quantitative PCR. Therefore, this study confirms that the overexpression of efflux pump genes plays an important role in the drug resistance of clinical isolates of *Mycobacterium tuberculosis*.

## INTRODUCTION

Tuberculosis was the primary cause of death from a single infectious disease before the COVID-19 pandemic. According to the World Heath Organization, there were approximate 10.6 million new cases of tuberculosis (TB) and 1.6 million deaths worldwide in 2021 (1). Additionally, drug-resistant TB is a significant public health concern. The highest burden of drug-resistant TB is observed in India (27%), China (14%), and the Russian Federation (8%) (2). In 2021, the burden of drug-resistant TB increased by 3%, with 450,000 new cases (1). In 2021, 3.6% of new TB cases and 18% of previously treated patients were found to be resistant to at least one anti-TB drug (1). At the same time, the COVID-19 pandemic has led to disruptions in the medical service of TB patients, resulting in a decrease in the number of people receiving continuous anti-TB treatment. This has increased the risk of drug-resistant TB. Cure rates for drug-resistant TB are much lower than those for drug-sensitive TB, with only 56% for multi-drug-resistant TB and 39% for extensively drug-resistant TB (3). The increased incidence of drug-resistant TB not only places a burden on global public health but also increases the economic pressure on patients and their families. Therefore, a comprehensive understanding of drug-resistance mechanisms is crucial for the design of new drugs and the development of alternative medicines.

It is believed that the drug resistance of *Mycobacterium tuberculosis* is the result of the synergetic effect of several mechanisms. The intrinsic antibiotic resistance of *Mycobacterium tuberculosis* (Mtb) to most antibiotics is often attributed to its cell wall, which has a special lipid-rich composition and structure that results in low drug permeability (4). In addition to the specific structure of Mtb, mutations in Mtb genomic regions or target genes that confer protection against anti-tuberculosis drugs are responsible for phenotypically diverse drug resistance (5). For example, mutations in genes such as *rpoB*, *katG*, *ahpC*, and *inhA* can cause drug resistance in Mtb (4, 6). While gene mutations are a frequent contributor to drug resistance, it should be noted that not all instances of drug resistance can be attributed to mutations in target genes alone. Previous research suggested that the efflux pumps could be alternative resistance mechanisms because not all phenotypically fluoroquinolone-resistant Mtb strains have canonical resistance mutations identified (7). Moreover, some studies conducting through bioinformatic analysis or by introducing or knocking out of the corresponding efflux pump genes in model organisms suggested that the efflux mechanism mediated by several proteins is also the main way to cause intrinsic drug resistance (8, 9). At the same time, studies have also shown that long-term exposure of Mtb to isoniazid (INH) can incite the efflux system, leading to an increase in the drug-resistant phenotype (10). However, there is still a lack of a large amount of clinical evidence to prove that the overexpression of efflux pump genes is common in drug-resistant clinical isolates.

Previous studies have confirmed that the efflux mechanism contributes to the intrinsic drug resistance of Mtb and other bacteria. It has been proposed that different families of efflux pumps are responsible for transporting different drugs. The five most representative families of efflux transport proteins identified in Mtb are the ATP-binding cassette/ABC superfamily, the major facilitator superfamily (MFS), the small multi-drug-resistance (SMR) family, the resistance-nodulation-cell division (RND) family, and the multi-drug and toxic compounds extrusion (MATE) family (11). Members of the MFS, SMR, RND, and MATE families are considered as secondary transporters and are usually energized by the proton motive force (H^+^ or Na^+^), while members of the ABC superfamily use ATP as an energy source and are considered as primary transporters. Narang et al. (12) found that INH-resistant isolates upregulated at least one of the genes *Rv1634*, *Rv0849*, *efpA*, and *p55* when exposed to INH. Similarly, Machado et al. (13) observed the overexpression of efflux pump genes *Mmr*, *mmpL7*, *Rv1258c*, *p55*, and *efpA* in the presence of antibiotics. Nevertheless, there is a lack of standard criteria for inter-comparison between different studies, including what internal reference genes should be selected and whether drug induction is necessary. Therefore, the results of different research groups may be quite inconsistent.

Efflux pump inhibitors are molecules that inhibit efflux pumps via one or more mechanisms, resulting in the deactivation of drug transport. For example, TriR acts as a repressor for a resistance-nodulation-cell division efflux pump TriABC, which is implicated in triclosan resistance in *Agrobacterium tumefaciens* (14). Moreover, NieR serves as the repressor of a NaOCl-inducible efflux system in *Agrobacterium fabrum* C58 (15). Efflux pump inhibitors have demonstrated their potential as combination drugs for anti-tuberculosis treatment. These molecules can inhibit the efflux of anti-tuberculosis drugs, improve drug efficacy, reverse resistance, and create synergistic effects when used with first-line anti-tuberculosis drugs. Verapamil has been shown to effectively inhibit drug efflux in previous studies (16, 17), and studies have demonstrated that the combination of verapamil with isoniazid or rifampicin (RIF) can reduce the MIC of INH or RIF and even reverse the resistance of Mtb.

Therefore, in this study, 46 clinical isolates, including 5 sensitive isolates, 5 RIF mono-resistant isolates, 18 INH mono-resistant isolates, and 18 multi-drug-resistant isolates, isolated from sputum specimens of inpatients with TB in Zigong, Sichuan, China, were selected for the experiment. The mutation sites of resistance relevant genes in the clinical isolates were analyzed by gene sequencing. Before investigating the efflux pump gene expression levels of the strains, we tested the stability of several internal reference gene candidates with or without drug exposure to screen the most stable reference genes for subsequent studies. Then, we determined the expressing levels of 10 putative efflux pump genes in isolated strains with or without drug exposure. The 10 genes are from four efflux transporter protein families, including SMR family (*Rv3065*), MATE family (*Rv2836c*), MFS family (*efpA*, *Rv1410c*, *Rv1250*, and *Rv0876c*), and ABC family (*Rv1819c*, *Rv0933*, and *Rv1217c-Rv1218c*). We also examined the drug resistance of the isolated strains after treating them with an efflux pump inhibitor to further understand the role of efflux pump genes in drug resistance of Mtb. Finally, we analyzed the correlation between molecular typing, drug-resistance gene mutations, and efflux gene overexpression.

## MATERIALS AND METHODS

### *Mycobacterium tuberculosis* clinical isolates

The 46 isolates of Mtb used in this study were isolated from sputum specimens of TB inpatients admitted to sentinel hospitals in Zigong, Sichuan, China, from 2018 to 2020. The standard strain H37Rv (ATCC 27294) was obtained from the Sichuan Center for Disease Control and Prevention (CDC). Forty-six clinical isolates were included in the study, consisting of 5 sensitive isolates, 5 RIF mono-resistant isolates, 18 INH mono-resistant isolates, and 18 multi-drug-resistant isolates. The isolated strains were cultured on Lowenstein-Jensen medium (Baso, China) at 37°C for 4–8 weeks for DNA extracting or were cultured using liquid medium, Middlebrook 7H9 (Becton Dickinson Biosciences, USA) medium containing 0.2% glycerol and 10% oleic albumin dextrose catalase (OADC) supplement (Hopebio, China) for 14 days for RNA extraction and MIC determination. The drug-sensitive phenotypes of the isolates were determined by the proportional drug-sensitivity test at the Zigong CDC. The specific drug-resistance information of the isolates is shown in Table S1. Our work was carried out under biosafety level (BSL)-2 containment with BSL-3 safety equipment and work practices.

### MIRU-VNTR typing

The bacteria colonies from a Lowenstein-Jensen medium were resuspended in 400-µL ddH_2_O and then heated in a metal bath at 98°C for 30 min, ultrasonically broken for 15 min (Elmasonic S 30, Germany), and centrifuged at 12,000 rpm for 3 min, and the supernatant was taken as the genomic DNA. The 12-locus MIRU-VNTR genotyping was carried out according to the description of a previous study (18). The bands were analyzed by 1.5% agarose gel electrophoresis.

### Determination of drug resistance-related gene mutations

The resistance determining regions of the *rpoB*, *KatG*, *inhA*, and *oxyR-ahpC* genes of clinical isolates were sequenced. The amplification primer sequences are listed in Table S2, and the amplification conditions are listed in Table S3. The amplification products were sent to a commercial sequencing company for sequence determination (Tsing Ke Biotechnology Co. Ltd, Chengdu, China). The sequence information of related genes was obtained from Mycobrowser (https://mycobrowser.epfl.ch/).

### Efflux pump gene expression assay

#### RNA extraction and cDNA synthesis

Total RNA was extracted using TRIzol reagent (Invitrogen, USA) (19). The integrity of the extracted RNA was verified using a 1% agarose gel, and the purity and content of the extracted RNA were determined using a NanoDrop 2000 Spectrophotometer (Thermo Fisher, USA) and a Qubit version 4.0 Fluorometer (Thermo Fisher). The RNA samples were diluted to 20 ng/µL with RNase-free ddH_2_O and stored at −80°C until use. cDNA was prepared by reverse transcription using HiScript III All-in-one RT SuperMix Perfect for quantitative PCR (qPCR) (R333-01, Vazyme, China). Before qPCR, the cDNA obtained was stored at −20°C.

#### Reference gene screening

For the screening of internal reference genes, we selected 15 strains including 5 sensitive isolates, 3 INH mono-resistant isolates, 2 RIF mono-resistant isolates, and 5 multi-drug-resistant isolates for the experiment. Each strain was cultured for 14 days before extracting the total RNA. Additionally, drug-resistant strains were cultured with the supplement of corresponding drug for 14 days, for example, one-half MIC RIF (Macklin, China) for RIF mono-resistant isolates, one-half MIC INH (Macklin) for INH mono-resistant isolates, and one-half MIC RIF, one-half MIC INH, or one-half MIC INH + one-half MIC RIF for the multi-drug-resistant isolates, respectively. Thus, a total of 35 total RNA samples were obtained for the test. The expression levels of eight candidate reference genes, *sigA*, 16S rRNA, *polA*, *secA*, *Hsp65*, GAPDH, *sigB*, and *rpoB*, were tested by qPCR. The q-PCR primer sequences for these eight genes are shown in Table S2. The data were analyzed using the following software tools: geNorm, NormFinder, ΔCT, Bestkeeper, and RefFinder (http://blooge.cn/RefFinder/).

#### qPCR assay

The reaction system included 10 µL of 2 × Taq Pro Universal SYBR qPCR Master Mix (Q712-02, Vazyme), 0.4 µL of primer forward (10 µM), 0.4 µL of primer reverse (10 µM), 1 µL of cDNA, and 8.2 µL of RNase-free ddH_2_O. The reaction conditions were 95°C for 30 s for once, 95°C for 10 s, and 60°C for 15 s for 40 cycles.

#### Determination of efflux pump gene expression levels before and after drug induction

After conducting a thorough literature review, we selected 10 specific efflux pump genes to be studied, including *Rv3065*, *Rv2836c*, *efpA*, *Rv1410c*, *Rv1250*, *Rv0876c*, *Rv1819c*, *Rv0933*, *Rv1217c*, and *Rv1218c*. The sequences of these 10 genes are available on the Mycobrowser (https://mycobrowser.epfl.ch/). The qPCR primer sequences for these 10 genes are shown in Table S2. To examine the impact of first-line drugs on efflux pump gene expression in drug-resistant isolates, the drug-resistant isolates were cultured with or without the corresponding drug induction. For drug induction, mono-resistant isolates were cultured on a medium supplemented with RIF or INH at one-half MIC for 14 days, and multi-drug-resistant isolates were cultured on medium supplemented with one-half MIC of RIF, one-half MIC of INH, or one-half MIC of INH + one-half MIC of RIF, respectively. The relative expression level was determined by the 2^−ΔΔCt^ method and based on the geometric mean of two reference genes selected from Reference Genes Screening. In this study, the efflux pump gene is considered to be overexpressed if the relative expression level is more than 4 and is considered as under-expressed if the relative expression level is less than 1 (20). If a strain has one overexpressed efflux pump gene, the strain is considered an efflux pump gene overexpression strain.

### Effect of efflux pump inhibitors

MIC determination was performed using the microplate alamar blue method, as described in the literature (21). The H37Rv strain (ATCC 27294) was used as a sensitive strain control. Firstly, the efflux pump inhibitor VP (MCE, China) was serially diluted twofold to a concentration ranging from 8 to 256 µg/mL, and its MIC was determined for all clinical isolates. Then the INH or RIF with Middlebrook 7H9 (Becton Dickinson Biosciences) medium containing 0.2% glycerol and 10% OADC (Hopebio, China) with or without one-half MIC of VP was serially diluted twofold to a concentration ranging from 0.001 to 128 µg/mL for INH) or from 0.001 to 256 µg/mL (for RIF), respectively, to determine the MICs of INH or RIF for all clinical isolates with or without the supplement of one-half MIC of VP. All tests were conducted twice. The MIC was defined as the lowest drug concentration which prevented a color change.

### Statistical analysis

The Kruskal-Wallis H rank-sum test was used to compare whether there was a difference in efflux pump gene expression between clinical isolates with different drug-resistance types (sensitive, RIF mono-resistant, INHmono-resistant, and multi-drug-resistant). One-sample *t*-tests for normally distributed samples and one-sample Wilcoxon signed rank-sum tests for non-normally distributed samples were performed to compare changes in efflux pump gene expression before and after drug induction. A *χ* ^2^ test was used to analyze the correlation between molecular typing, gene mutations, and efflux pump gene overexpression. All statistical analyses were performed in IBM SPSS version 26.0 software. A *P* value of <0.05 was specified as statistically significant.

## RESULTS

### Strain genotyping and drug-resistance gene mutations

As shown in Table S4, 80% (four of five) of the mono-resistant strains included in the study showed mutations in the *rpoB* gene. Among them, the molecular typing results of MIRU-VNTR for RR-1 (mutant type) and RR-5 (wild type) are the same. Among 18 strains of INH-resistant strains, 77.8% (14 of 18) detected mutations in INH-related resistance genes. Among them, *katG* (315 AGC → ACC) is the most common mutation type, accounting for 71.4% (10 of 14) of the mutant strains, followed by *inhA* (−15C → T), accounting for 35.7% (5 of 14) of the mutant strains. The HR-9 strain contains *katG* (315 AGC → ACC) and *inhA* (−15C → T) double site mutation. The MIRU-VNTR typing results of 18 INH mono-resistant strains were different. Eighteen multi-drug-resistant strains all had mutations in the resistance genes. *rpoB* (531 TCG → TTG) and *katG* (315 AGC → ACC) are the main mutation types, accounting for 61.1% (11 of 18) and 100% (18 of 18), respectively. The two site mutations in multi-drug-resistant strains mainly focus on the *rpoB* gene, including *rpoB* on MDR-1 (516 TGG → TGT/517 ACC → TCC), *rpoB* on MDR-11 (511 CTG → CCG/512 AGC → GGC), and *rpoB* on MDR-15 (511 CTG → CCG/572 ATC → CTC). The MIRU-VNTR typing results of 18 multi-drug-resistant strains were different.

### Screening of reference genes

The screening results showed that the Ct values of all candidate reference genes ranged from 23.720 [16S rRNA, standard deviation (SD) ±3.720] to 31.466 (*polA*, SD ±3.087). The geNorm stability ranking result was *secA* = *sigA* > *Hsp65* > *GAPDH* > *sigB* > *rpoB* > *polA* > 16S rRNA; the NormFinder stability ranking result was *secA* > *Hsp65* > *GAPDH* > *rpoB* > *sigA* > *sigB* > *polA* > 16S rRNA; the BestKeeper stability ranking result was *sigA* > *rpoB* > *secA* > *GAPDH* > *Hsp65* > *sigB* > *polA* > 16S rRNA; the ΔCT analysis stability ranking result was *secA* > *Hsp65* > *GAPDH* > *sigA* > *rpoB* > *sigB* > *polA* > 16S rRNA; and the RefFinder stability ranking result was *secA* > *sigA* > *Hsp65* > *GAPDH* > *rpoB* > *sigB* > *polA* > 16S rRNA. Based on the above software analysis, *secA* and *sigA* were identified as the most stable expressed reference genes for subsequent experiment on the expression level of efflux pump genes.

### Expression levels of efflux pump genes in different drug-resistant types of isolates

It was found that all the selected 10 efflux pump genes did not overexpress in sensitive strains, but overexpression was observed in some drug-resistant strains. However, not all drug-resistant strains are efflux pump gene overexpression strains. One hundred percent (5 of 5) of RIF mono-resistant strains, 44.4% (8 of 18) of INH mono-resistant strains, and 88.9% (16 of 18) of multi-drug-resistant strains are efflux pump gene overexpression strains (Tables 1 through 3).

**TABLE 1:** Overexpressed efflux pump genes in RIF mono-resistant isolates before and after drug induction^*a*^

Sample ID	No drug induction	Efflux pump gene overexpression strain	One-half MIC of RIF induction
RR-1	*Rv2836*, *Rv1250*, *Rv1819c*, *Rv0933*, and *Rv1217c*	Y	*Rv2836*, *Rv1250↑*, *Rv1819c*, *Rv0933*, and *Rv1217c*
RR-2	*Rv3065*, *Rv1410c*, *Rv1250*, and *Rv1819c*	Y	*Rv3065↑*, *Rv1410c*, *Rv1250↑*, *Rv0876c*, *Rv1819c*, *Rv0933*, and *Rv1217c*
RR-3	*Rv3065* and *Rv1819c*	Y	*Rv3065↑*, *Rv1410c*, *Rv1250↑*, *Rv0933*, and *Rv1217c*
RR-4	*Rv3065*, *Rv2836*, *efpA*, *Rv1410c*, *Rv1250*, *Rv0876c*, *Rv1819c*, *Rv0933*, and *Rv1217c*	Y	*Rv3065↑*, *Rv2836*, *efpA*, *Rv1410c*, *Rv1250↑*, *Rv0876c*, *Rv1819c*, *Rv0933*, and *Rv1217c*
RR-5	*Rv3065*, *Rv1410c*, *Rv1250*, *Rv1819c*, and *Rv1217c*	Y	*Rv3065↑*, *Rv2836*, *efpA*, *Rv1410c*, *Rv1250↑*, *Rv0876c*, *Rv1819c*, *Rv0933*, and *Rv1217c*

^*a*^ RR represents RIF mono-resistant strains. Y means Yes. "↑" indicates significantly increased relative expression level of genes after drug induction (*P* < 0.05).

**TABLE 2:** Overexpressed efflux pump genes in INH mono-resistant isolates before and after drug induction^*a*^

Sample ID	No drug induction	Efflux pump gene overexpression strain	One-half MIC of INH induction
HR-1	*/*	N	*/*
HR-2	*Rv1250*	Y	*/*
HR-3	*Rv1250* and *Rv0933*	Y	*Rv3065*, *Rv2836*, *Rv1250*, *Rv0933*, *Rv1217c*, and *Rv1218c*
HR-4	*Rv0933*	Y	*Rv0933*
HR-5	*/*	N	*/*
HR-6	*Rv0933*	Y	*/*
HR-7	*/*	N	*Rv1218c*
HR-8	*Rv0933*, *Rv1217c*, and *Rv1218c*	Y	*Rv1250* and *Rv0933*
HR-9	*Rv2836*, *Rv1250*, *Rv0933*, *Rv1217c*, and *Rv1218c*	Y	*/*
HR-10	*Rv3065*, *Rv1250*, and *Rv0933*	Y	*/*
HR-11	*/*	N	*Rv3065* and *Rv0933*
HR-12	*/*	N	*/*
HR-13	*/*	N	*/*
HR-14	*/*	N	*/*
HR-15	*/*	N	*/*
HR-16	*/*	N	*/*
HR-17	*Rv0933*	Y	*/*
HR-18	*/*	N	*/*

^*a*^ HR represents INH mono-resistant strains. Y means Yes. N means No. "/" means that none of the 10 efflux pump genes is overexpressed.

**TABLE 3:** Overexpressed efflux pump genes in multi-drug-resistant isolates before and after drug induction^*a*^

Sample ID	No drug induction	Efflux pump gene overexpression strain	Drug induction		
			One-half MIC of RIF	One-half MIC of INH	Obe-half MIC of RIF + one-half MIC of INH
MDR-1	*Rv3065*, *efpA*, *Rv1410c*, *Rv1250*, *Rv0933*, and *Rv1217c*	Y	*Rv1250, Rv0933↓, Rv1217c↓*	***/***	*Rv1250↓*
MDR-2	*Rv1250* and *Rv0933*	Y	***/***	***/***	*Rv3065*, *Rv1250↓*, and *Rv0933*
MDR-3	*Rv2836*, *Rv1250*, and *Rv0933*	Y	***/***	*Rv1218c↑*	*Rv1410c*
MDR-4	*Rv3065*, *Rv2836, Rv1410c*, *Rv1250*, and Rv0933	Y	***/***	*Rv3065*, *Rv2836*, *Rv1250↓*, *Rv0876c↓*, *Rv0933↓*, and *Rv1217c*	*Rv1250↓* and *Rv0933*
MDR-5	*Rv2836*, *Rv1250*, *Rv0876c*, *Rv0933*, and *Rv1217c*	Y	***/***	***/***	*Rv0933*
MDR-6	*Rv3065*	Y	***/***	***/***	*Rv0933*
MDR-7	*Rv2836*, *efpA*, *Rv1250*, *Rv0876c*, *Rv0933*, *Rv1217c*, and *Rv1218c*	Y	***/***	*Rv1250↓*, *Rv0933↓*, *Rv1217c*, and *Rv1218c↑*	*Rv1250↓*
MDR-8	*Rv2836*, *Rv1250*, *Rv0876c*, *Rv0933*, and *Rv1217c*	Y	*Rv2836↓* and *Rv1250*	*Rv2836*, *Rv1250↓*, *Rv0933↓*, and *Rv1217c*	*Rv3065*, *Rv1410c*, *Rv1250↓*, and *Rv0933*
MDR-9	*Rv3065*, *Rv1250*, and *Rv0933*	Y	*Rv0933↓*	*Rv2836*, *efpA*, *Rv1250↓*, *Rv0933↓*, and *Rv1217c*	***/***
MDR-10	*Rv3065*, *Rv2836*, and *Rv1250*	Y	*Rv3065↓*, *Rv1250*, and *Rv0933↓*	*Rv1250↓* and *Rv0933↓*	*Rv3065*, *Rv1410c*, *Rv1250↓*, and *Rv0933*
MDR-11	*Rv1250* and *Rv0933*	Y	*Rv1250* and *Rv0933↓*	***/***	***/***
MDR-12	*Rv1250* and *Rv0933*	Y	***/***	***/***	***/***
MDR-13	***/***	N	***/***	***/***	***/***
MDR-14	*Rv2836*, *Rv1250*, *Rv0933*, and *Rv1217c*	Y	***/***	*Rv1250↓* and *Rv0933↓*	***/***
MDR-15	***/***	N	*Rv2836↓* and *Rv1250*	*Rv2836* and *Rv1250↓*	*Rv2836* and *Rv1250↓*
MDR-16	*Rv1250*	Y		*Rv1250↓*	*Rv1250↓*
MDR-17	*Rv3065*, *efpA*, *Rv1410c*, *Rv0876c*, *Rv0933*, and *Rv1217c*	Y	*Rv3065↓*, *Rv1410c↓*, *Rv1250*, *Rv0876c↓*, *Rv0933↓*, and *Rv1217c↓*	*Rv3065*, *Rv1410c*, *Rv1250↓*, *Rv0876c↓*, and *Rv0933↓*	*Rv3065*, *Rv1410c*, and *Rv0933*
MDR-18	*Rv3065*, *Rv1410c*, *Rv0933*, and *Rv1217c*	Y	*Rv3065↓*, *Rv1410c↓*, *Rv1250↓*, and *Rv0933↓*	*Rv3065*, *Rv1250↓*, and *Rv0933↓*	*Rv3065*

^*a*^ MDR represents multi-drug resistant strains. Y means Yes. N means No. "/" means that none of the 10 efflux pump genes is overexpressed.？"↑" indicates significantly increased relative expression level of genes after drug induction (*P* < 0.05), and "↓" indicates significantly decreased relative expression level of genes after drug induction (*P* < 0.05).

**Fig 1:**
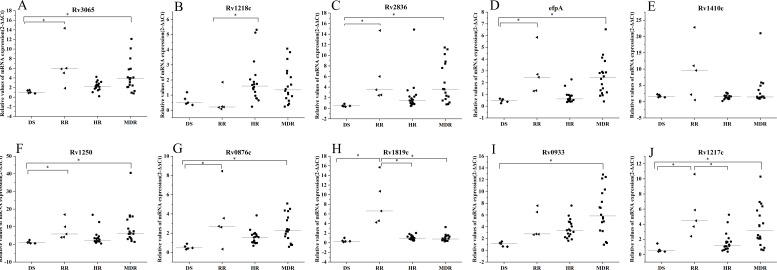
Relative expression levels of 10 efflux pump genes among different drug-resistant types of isolates. *P < 0.05, Kruskal-Wallis H rank-sum test. The graphs A–I and J show scatter plots of the relative expression levels of *Rv3065*, *Rv2836c*, *efpA*, *Rv1410c*, *Rv1250*, *Rv0876c*, *Rv1819c*, *Rv0933*, *Rv1217c*, and *Rv1218c* in different drug-resistant types of strains, respectively; DS represents sensitive strains; RR represents RIF mono-resistant strains; HR represents INH mono-resistant strains, and MDR represents multi-drug-resistant strains.

Analysis of the relative expression levels of efflux pump genes in different drug-resistant types of isolates revealed that the relative expression levels of six genes, *Rv3065*, *Rv2836c*, *efpA*, *Rv1250*, *Rv0876c*, and *Rv1217c*, were statistically different in RIF mono-resistant and multi-drug-resistant isolates compared to sensitive isolates, while the relative expression levels in INH mono-resistant strains were not statistically different compared to sensitive strains (Fig. 1A through C, E, F and I). The relative expression level of the *Rv0933* gene in multi-drug-resistant isolates was statistically different compared with that in sensitive isolates (Fig. 1H); the relative expression level of *Rv1218c* gene in INH mono-resistant isolates was higher than that in RIF mono-resistant isolates (Fig. 1J), while the relative expression level of *Rv1217c* gene in RIF mono-resistant isolates was higher than that in INH mono-resistant isolates (Fig. 1I); the relative expression level of *Rv1819c* gene was higher in RIF mono-resistant isolates than in the other three types of isolates (Fig. 1G), while the relative expression level of *Rv1410c* gene was not statistically different in different resistant types of isolates (Fig. 1D).

**Fig 2:**
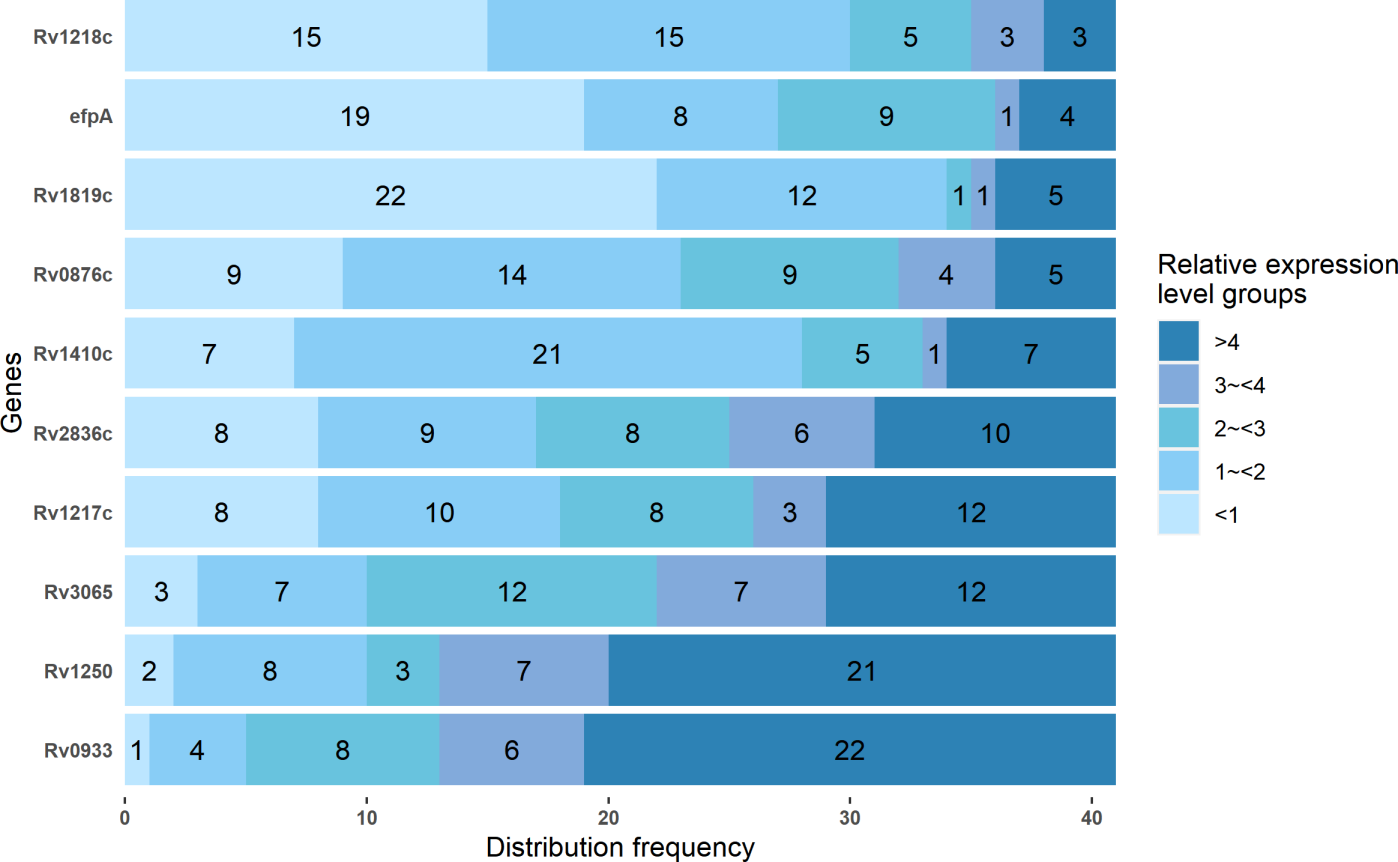
Distribution frequency of relative expression levels of 10 efflux pump genes among different level groups. Different colors represent different relative expression levels. The numbers in the bars of the graph indicate the number of strains with the specified relative expression levels of 10 efflux pump genes.

As shown in Fig. 2, of the 41 resistant isolates, *Rv1250* (51.2%, 21 of 41) and *Rv0933* (53.7%, 22 of 41) showed a higher distribution frequency of overexpression (relative expression level >4), while *efpA* (46.3%, 19 of 41), and Rv1819c (53.7%, 22 of 41) showed higher frequency of under-expression (relative expression level <1), the relative expression levels of other genes were relatively evenly distributed among each relative expression level group.

Among RIF mono-resistant isolates, *Rv1819c* (100%, five of five) is the gene with the highest frequency of overexpression (Table 1). Among INH mono-resistant isolates, *Rv0933* (38.9%, 7 of 18) is the gene with the highest frequency of overexpression (Table 2). Among multi-drug-resistant strains, *Rv1250* (72.2%, 13 of 18) and *Rv093*3 (72.2%, 13 of 18) are both the genes with the highest frequency of overexpression (Table 3).

### Effects of drug induction on efflux pump gene expression

Determination of the relative expression levels of putative efflux pump genes in drug-resistant isolates under the administration of drug stress revealed that, after RIF induction, the numbers of overexpressed efflux pump genes increased in 60% (three of five) of the RIF mono-resistant isolates (Table 1); but in INH mono-resistant isolates (Table 2), there was no definite change trend in the number of overexpressed efflux pump genes after INH induction. Some isolates showed more overexpressed efflux pump genes after induction, but some isolates showed less overexpressed genes, and some isolates showed no change in the number of overexpressed genes before and after drug induction. For example, four efflux pump genes became overexpressed (relative expression level >4) in isolate HR-3 after INH induction, while in isolates HR-9 and HR-10, previously overexpressed efflux pump genes became no longer overexpressed after INH induction (Table 2). In multi-drug-resistant isolates (Table 3), similar to INH mono-resistant isolates, there was no definite change trend in the number of overexpressed efflux pump genes after drug induction.

A one-sample *t*-test for normally distributed samples and a one-sample Wilcoxon signed rank-sum test for non-normally distributed samples showed an increase in the relative expression levels of *Rv3065* (*t* = 3.115, *P* < 0.05) and *Rv1250* (*t* = 2.899, *P* < 0.05) after one-half MIC of RIF induction in mono-RIF-resistant isolates. In mono-resistant INH isolates, no significant changes in the relative expression levels of all 10 efflux pump genes were observed after one-half MIC of INH induction (*P* > 0.05). In multi-drug-resistant isolates, the relative expression levels of *Rv1250* (*w* = −0.653, *P* > 0.05) and *Rv1218c* (*t* = 0.567, *P* > 0.05) did not show significant changes after one-half MIC of RIF induction, while the relative expression levels of the remaining efflux pump genes decreased; the relative expression levels of *Rv1250* (*w* = −2.613, *P* < 0.05), *Rv0876* (*w* = −1.960, *P* < 0.05), and *Rv0933* (*t* = −2.866, *P* < 0.05) showed significant decrease, and the relative expression level of *Rv1218c* (*t* = 2.326, *P* < 0.05) increased after one-half MIC of INH induction; after one-half MIC of RIF and one-half MIC of INH co-induction, the relative expression levels of *Rv1250* (*t* = −6.179, *P* < 0.05), *Rv1819c* (*w* = −2.961, *P* < 0.05), and *Rv1218c* (*w* = −2.047, *P* < 0.05) decreased, and the remaining efflux pump genes did not show significant changes.

### MIC results before and after the intervention of the efflux pump inhibitor

The effects of the efflux pump inhibitor VP on the MICs of the drug-resistant isolates are compared in Table 4. INH MICs for five sensitive clinical isolates and H37Rv ranged from 0.0156 to 0.0625 μg/mL, and RIF MICs ranged from 0.0156 to 0.0625 μg/mL as well (Table 4). After the efflux pump inhibitor VP intervention, the INH MICs decreased from 0.0075 to 0.0312 µg/mL and the RIF MICs decreased from 0.0075 to 0.0156 µg/mL (Table 4). The RIF MICs for RIF mono-resistant isolates ranged from 1 to 32 μg/mL, and the RIF MICs decreased from 0.25 to 16.0 µg/mL after the intervention of the efflux pump inhibitor VP (Table 4). The INH MIC for INH mono-resistant isolates was 0.5–4.0 µg/mL, and the range was unchanged after the intervention of VP (Table 4). The INH MICs for multi-drug-resistant isolates ranged from 0.5 to 64.0 µg/mL, and the RIF MICs ranged from 0.5 to 256.0 µg/mL; the INH MICs decreased from 0.25 to 32 µg/mL, and the RIF MICs decreased to from 0.5 to 128 µg/mL after the intervention of VP (Table 4).

**TABLE 4:** MICs before and after the intervention of the efflux pump inhibitor^*a*^

Number	INH MICs (μg/mL)	INH + VP MICs (μg/mL)	*P* value	RIF MICs (μg/mL)	RIF + VP MICs (μg/mL)	*P* value
H37Rv	0.0625	**0.0312**	*t* = 7.000, *P =* 0.001	0.0625	**0.0075**	*t* = 3.000, *P* = 0.030
DS-1	0.0156	**0.0075**		0.0156	**0.0075**	
DS-2	0.0156	**0.0075**		0.0156	**0.0075**	
DS-3	0.0156	**0.0075**		0.0156	**0.0075**	
DS-4	0.0156	**0.0075**		0.0156	**0.0075**	
DS-5	0.0312	**0.0075**		0.0312	**0.0156**	
RR-1	**/**	**/**	**/**	32	**16**	*t* = 3.500, *P* = 0.025
RR-2	**/**	**/**		32	**8**	
RR-3	**/**	**/**		2	2	
RR-4	**/**	**/**		32	**8**	
RR-5	**/**	**/**		1	**0.25**	
HR-1	4	4	**/**	**/**	**/**	**/**
HR-2	4	4		**/**	**/**	
HR-3	4	4		**/**	**/**	
HR-4	0.5	0.5		**/**	**/**	
HR-5	2	2		**/**	**/**	
HR-6	4	4		**/**	**/**	
HR-7	2	2		**/**	**/**	
HR-8	1	1		**/**	**/**	
HR-9	4	4		**/**	**/**	
HR-10	2	2		**/**	**/**	
HR-11	4	4		**/**	**/**	
HR-12	0.5	0.5		**/**	**/**	
HR-13	1	1		**/**	**/**	
HR-14	1	1		**/**	**/**	
HR-15	0.5	0.5		**/**	**/**	
HR-16	4	4		**/**	**/**	
HR-17	2	2		**/**	**/**	
HR-18	4	4		**/**	**/**	
MDR-1	2	2	*t* = 2.715, *P* = 0.015	2	**0.5**	*t* = 3.117, *P* = 0.006
MDR-2	2	2		64	**32**	
MDR-3	2	2		256	**128**	
MDR-4	16	**8**		256	**64**	
MDR-5	2	**1**		256	**64**	
MDR-6	2	2		32	**16**	
MDR-7	2	2		16	16	
MDR-8	64	**16**		256	**16**	
MDR-9	32	**16**		128	**64**	
MDR-10	2	2		128	**64**	
MDR-11	16	**8**		64	64	
MDR-12	64	**32**		32	**16**	
MDR-13	0.5	**0.25**		16	**4**	
MDR-14	1	1		32	**8**	
MDR-15	1	1		1	1	
MDR-16	2	2		0.5	0.5	
MDR-17	2	2		8	**4**	
MDR-18	2	2		1	1	

^*a*^ DS represents sensitive strains, RR represents RIF mono-resistant strains, HR represents INH mono-resistant strains, and MDR represents multi-drug resistant strains. "/" means that none of the 10 efflux pump genes is overexpressed. The MICs reduced after the intervention of the efflux pump inhibitor VP are highlighted in bold.

The log2 values of the fold decrease in MICs before and after the intervention of VP were subjected to independent sample *t*-test to compare the effect of VP in different resistant types of isolates, and the results showed that the efflux pump inhibitor VP had no effect on the INH MICs for mono-resistant INH isolates but significantly reduced the INH and RIF MICs for sensitive isolates (INH: *t* = 7.000, *P* = 0.001; RIF: *t* = 3.000, *P* = 0.030) and RIF MICs for mono-resistant RIF isolates (RIF: *t* = 3.500, *P* = 0.025), and also reduced INH and RIF MICs for multi-drug-resistant isolates (INH: *t* = 2.715, *P* = 0.015; RIF: *t* = 3.117, *P* = 0.006).

### Relationship between polymorphic loci, drug-resistance gene mutations, and efflux pump gene overexpression

In the phenotypic surveillance of past drug-resistant isolates in Zigong, two loci, MIRU26 and MIRU31, were found to be highly polymorphic among the 12 typing loci of MIRU-VNTR. It is suspected that there may be some correlation between the polymorphic loci with the overexpression of the efflux pump genes. In addition, it has been pointed out in some literature that overexpression of efflux pump genes may be a compensatory resistance mechanism for isolates without resistance gene mutations or an intermediate link before the occurrence of resistance gene mutations. As shown in Table 5, in this study, by comparing the incidence of efflux pump gene overexpression in different MIRU-VNTR isolates, it was found that the two most polymorphic loci, MIRU26 (*χ* ^2^ = 0.043, *P* > 0.05) and MIRU31 (*χ* ^2^ = 1.406, *P* > 0.05), were not associated with the incidence of efflux pump gene overexpression, whereas strains that had drug-resistance gene mutations showed a higher rate of efflux pump gene overexpression than isolates without mutations (*χ* ^2^ = 7.241, *P* = 0.007).

**TABLE 5:** Correlation analysis between factors and the incidence of efflux pump gene overexpression

Factors	Numbers of efflux pump gene overexpression strains	Numbers of total strains	*χ* ^2^	*P*
MIRU26			0.043	0.836
Repetitions (≤6）	12	20		
Repetitions (>6）	17	27		
MIRU31			1.406	0.236
Repetitions (≤3）	8	16		
Repetitions (>3）	21	31		
Mutation			7.241	0.007
No mutations in related drug-resistance genes have occurred.	2	10		
Mutations in related drug-resistance genes have occurred	27	37		

## DISCUSSION

In this study, the expression levels of efflux pump genes were determined by the 2^−ΔΔCt^ method. Selecting stably expressed reference genes during data analysis is the basis of research, and it is crucial for standardizing experimental data. However, it has been found that no reference gene is “universal” under all experimental conditions, and using only one reference gene is subject to relative error (22). Moreover, there is no unified internal reference gene for determining the efflux pump gene expression levels in Mtb clinical strains until now. The selection of different reference genes is likely to result in incomparability between similar studies. Therefore, in this study, firstly, we conducted the experiment to screen the most stable reference genes and then carried out the subsequent studies based on the two reference genes screened out.

The results showed that the abundance of the candidate internal reference genes varied, with Ct values ranging from 23.720 (16S rRNA, SD ±3.720) to 31.466 (*polA*, SD ±3.087). GeNorm, NormFinder, ΔCT, and Bestkeeper are four software applications that differ in their calculation mode, resulting in slightly different ranking results. The geNorm software is suitable for the calculation of small samples of paired genes but favors selection of inter-related genes (e.g., expressed in the same pathway). NormFinder has the advantage of distinguishing between intra-group and inter-group variation and is therefore suitable for evaluating candidate genes in different sample groups, but it requires a larger sample size (*n* > 8) compared to geNorm (23). To solve the problem of the inconsistent results obtained by the above different software applications, Xie et al. (24) integrated these four analysis methods into one network analysis tool, RefFinder, to evaluate the results of the above four software applications in a comprehensive manner. In this study, RefFinder rated *secA* and *sigA* as the top two most stable genes, and both *secA* and *sigA* genes were ranked as the most stable genes among the other four software applications. The MIQE Guidelines: Minimum Information for Publication of Quantitative Real-Time PCR Experiments (25) emphasizes the importance of using multiple reference genes for standardization when there is no sufficient evidence to prove the use of a single reference gene. Therefore, in this study, we decided to select both *secA* and *sigA* as reference genes for the subsequent determination of relative expression levels of efflux pump genes.

Regarding the expression of efflux pump genes, the following four main questions were explored in this study.

The first question is whether overexpression of efflux pump genes is specific to the resistant strains. The answer is yes. Overexpression of the selected 10 efflux pump genes was not observed in the sensitive strain in this study, which is consistent with the research of Gupta et al. (26). However, some studies have reported overexpression of efflux pump genes in sensitive isolates (12). There could be two reasons for the discrepancy: firstly, the efflux pump genes selected are different between the studies; secondly, the judgment standard for overexpression is different; for example, the relative expression level >2.5 is defined as overexpression.

The second question is whether overexpression of the efflux pump gene is associated with drug induction. The answer may well be no. The results of this study showed that in some drug-resistant clinical isolates, overexpression of the efflux pump gene occurs in the absence of drug induction, while the relative expression levels decrease after induction with drugs. It can be inferred that overexpression of the efflux pump genes is an inherent characteristic in drug-resistant clinical isolates, and similar phenomena have been observed in previous studies (27) and in other bacteria (28).

The third question is whether overexpression of efflux pump genes is associated with mutations in drug resistance-related genes and genotypes. In this study, two MIRU-VNTR polymorphic loci, MIRU26 and MIRU31, were selected to investigate the correlation between polymorphic loci and overexpression, and the results showed that there was no association between them. It has been suggested (12) that overexpression of the efflux pump gene is more likely to occur in isolates that do not have resistance-associated gene mutations, acting as a compensatory mechanism. However, in this study, isolates with resistance-associated gene mutations were more likely to overexpress the efflux pump genes. So it is suggested that resistance-associated gene mutations and efflux pump gene expression are not entirely independent, and there may be a synergistic relationship between them. However, in RR-5 strains which had no *rpoB* gene mutations, the number of overexpressed efflux pump genes was not significantly higher than other strains that had gene mutations, and in some INH mono-resistant strains that had no gene mutations, overexpression of any of the selected efflux pump genes was not even found. Therefore, it is still doubtful that the overexpression of efflux pump genes can explain the drug resistance of all strains without resistance-associated gene mutations.

The fourth question is how effective the efflux pump inhibitor VP is and whether there is any prospect of its application. In some previous studies, it was noted that when VP was used in combination with anti-tuberculosis drugs [including first- and second-line drugs (11, 29, 30) as well as some newly developed new drugs (31)], it showed significant effects both *in vivo* and *in vitro*. However, in this study, VP did not have a significant effect on INH mono-resistant isolates, which is consistent with the results of previous studies (17). However, in contrast, other studies (12, 17) pointed out that VP reduces the INH MICs for almost all INH-resistant strains. Possible explanations for such contradictory results are that the efflux pump genes did not show significant overexpression in the clinical isolates in this study, so that VP did not show significant effect to reverse bacterial resistance. In this study, VP reduced the RIF MICs of most of the selected isolates, which is consistent with previous studies (29).

The discovery of efflux pump drug-resistance mechanism provides a promising therapeutic approach to shorten the duration of TB treatment. The use of efflux pump inhibitors not only inhibits bacterial growth but also reduces bacterial resistance to drugs. Although the use of VP did not have the intended effect on INH mono-resistant isolates in this study, it did significantly decrease the drug MICs for RIF mono-resistant and multi-drug-resistant isolates. Therefore, the results suggest that VP may be a possible approach to be combined with anti-tuberculosis drug for the treatment of rifampicin-resistant tuberculosis/multi-drug resistant tuberculosis and drug-sensitive TB to enhance the efficacy and shorten the duration of drug treatment. However, despite the positive results of *in vitro* studies, including our own, the current clinic trial evidence is insufficient to support the use of VP as a true antibacterial co-agent. *In vivo* trials (30, 32) have shown that VP can affect the transmembrane proton motive force, which may not only affect bacteria but also interfere with the host cell. Despite the limitations of the clinical application of VP, efflux pump inhibitors with low toxicity to humans are still the direction for new drug development. For example, a new efflux pump inhibitor, 2-aminothia-zole UPAR-174, can dissipate membrane potential and cause ATP depletion at lower concentrations (33). In addition to using drugs that workable at low doses, it is important to find more specific drugs that only bind to the efflux pump of Mtb (34).

Ten efflux pump genes were selected in this study, which are from four efflux transporter protein families including SMR family (*Rv3065*), MATE family (*Rv2836c*), MFS family (*efpA*, *Rv1410c*, *Rv1250*, and *Rv0876c*) and ABC family (*Rv1819c*, *Rv0933*, and *Rv1217c-Rv1218c*).

The *Mmr* (*Rv3065*) is the only member of the SMR family so far reported to be associated with drug resistance in Mtb. Its substrates include acridin, ethidium bromide, fluoroquinolones, INH and macrolides, but it can be inhibited by compounds such as quercetin, reserpine, powdered antibiotics, and VP (11). Previous studies (27, 35) have shown that clinical isolates developed INH resistance through long-term exposure to INH, and at the same time, the isolates upregulated *Mmr* (*Rv3065*) gene expression. This suggested a possible association between *Mmr* (*Rv3065*) and INH resistance. Meanwhile, overexpression of *Rv3065* has been reported in multi-drug-resistant clinic isolates (36). In this study, the role of *Rv3065* in RIF resistance was confirmed as *Rv3065* was overexpressed in 80% (four of five) of the RIF mono-resistant isolates, and the expression level was upregulated after RIF induction in every RIF mono-resistant isolate, as well as in the multi-drug-resistant isolates. In contrast, *Rv3065* did not show significant overexpression in the INH mono-resistant isolates either before or after induction. Contrary to our study, Gupta et al. (37) and Pang et al. (38) did not find overexpression of the *Rv3065* in clinical strains isolating from drug-resistant TB after RIF induction.

*DinF* (*Rv2836c*) is considered by Mishra and Daniels to be the only MATE family transporter protein. So far, there is little research on *Rv2836c*. One study predicted that it should have 12 transmembrane structural domains (39). The structure and mechanism of *Rv2836c* have not yet been extensively researched; however, the present study indicates that overexpression of *Rv2836c* in mono-resistant and multi-drug-resistant isolates may contribute to their resistance to both INH and RIF.

Among the numerous efflux pump genes, the MFS and ABC superfamilies contain abundant family members, and previous studies have shown a correlation between them and drug resistance.

Our study selected genes from the MFS superfamily, including *efpA*, *Rv1410c*, *Rv1250*, and *Rv0876c*. Previous studies have reported the overexpression of *efpA* (20, 35, 40), *Rv1410c* (20, 36), and *Rv1250* (20) in clinic drug-resistance isolates, but there are almost no similar reports about *Rv0876c*. In this study, all four genes showed overexpression in drug-resistant strains.

In 2000, Braibant et al. (8) predicted the genes encoding the ABC superfamily transporter protein in the Mtb genome through sequence analysis. They believed that the genes encoding the ABC transporters accounted for approximately 2.5% of the Mtb genome. Moreover, they predicted the types of drugs that the related transporter members could transport. At the same time, studies have shown that the efflux pump genes of the ABC family also mediate the interaction between hosts and pathogens, which has a profound impact on prolonging the duration of chronic infections and exacerbating the final outcome of infections (41). Therefore, the role played by the ABC superfamily in drug resistance has been the focus of attention in efflux pump-related studies. *Rv1819c* (42), *Rv0933* (38), and *Rv1217c-Rv1218c* (43) selected from the ABC superfamily in this study have all been reported to be overexpressed in drug-resistance strains or have higher expression level in drug-resistance strains than in sensitive strains. In this study, *Rv1819c*, *Rv0933*, and *Rv1217c-Rv1218* were also overexpressed in drug-resistant strains, which is consistent with previous studies.

The research results also indicated that *Rv1819c* and *Rv0933* are the genes with the highest frequency of overexpression in RIF mono-resistant strains and INH mono-resistant strains, respectively, suggesting that the ABC family may play a major role in mono-resistant strains. Among multi-drug resistant strains, *Rv1250* and *Rv0933* are both genes with the highest frequency of overexpression, indicating that the MFS and ABC families may play a significant role in Mtb multi-drug resistance.

The differences in overexpression results between this study and some similar studies can be attributed to variations in bacterial genetic background, exposure time, and concentration of the drug, as well as the selection of internal reference genes. Due to the lack of a uniform standard, the performance of efflux pump genes can only be observed under specific conditions in different studies. The results of different studies cannot be completely compared with each other and cannot be generalized to all resistant strains. To address this issue, it is crucial to establish a unified standard for investigating the role of efflux pump genes in Mtb drug resistance. This study improved the reliability of mRNA expression level analysis by screening stable double reference genes, which can be used as a reference for future research.

This study considers that both gene mutations and efflux pump overexpression are factors for Mtb resistance. However, this study neither fully demonstrates the synergistic effect between the two mechanisms nor confirms their temporal relationship. Previous studies (27, 35) tried to investigate whether overexpression of efflux pump genes preceded gene mutation in the evolution of drug-resistant strains over a longer period of time by long-term exposure of the sensitive strains to subinhibitory concentrations of drugs. However, due to the complexity of the host *in vivo* environment, considering only drug pressure is not comprehensive enough. Therefore, comprehensive *in vivo* tests are necessary to verify these findings.

### Conclusion

Resistance cannot be attributed to a single mechanism. Overexpression of the efflux pump genes and resistance-related gene mutations together constitute drug resistance in Mtb. Inhibition of the efflux pump may provide a new adjuvant treatment option for patients with drug-resistant TB.

## SUPPLEMENTAL MATERIAL

Supplemental material spectrum.02510-23-s0001.docx Tables S1 to S4.

ASM does not own the copyrights to Supplemental Material that may be linked to, or accessed through, an article. The authors have granted ASM a non-exclusive, world-wide license to publish the Supplemental Material files. Please contact the corresponding author directly for reuse.
