# Supplementary material for: Overexpression of efflux pump genes is one of the mechanisms causing drug resistance in Mycobacterium tuberculosis
Source: Microbiol Spectr. 2023 Dec 4;12(1):e02510-23. doi: 10.1128/spectrum.02510-23 (PMC10783012; doi:10.1128/spectrum.02510-23)
Supplement: Supplemental material — Tables S1 to S4. [file spectrum.02510-23-s0001.docx]

**Table S1 Drug-sensitive phenotypes of the isolates**

| Number | sample ID | Drug resistance |
| --- | --- | --- |
| 1 | H37Rv | susceptible |
| 2 | DS-1 | susceptible |
| 3 | DS-2 | susceptible |
| 4 | DS-3 | susceptible |
| 5 | DS-4 | susceptible |
| 6 | DS-5 | susceptible |
| 7 | RR-1 | RIF |
| 8 | RR-2 | RIF |
| 9 | RR-3 | RIF |
| 10 | RR-4 | RIF |
| 11 | RR-5 | RIF |
| 12 | HR-1 | INH |
| 13 | HR-2 | INH |
| 14 | HR-3 | INH |
| 15 | HR-4 | INH |
| 16 | HR-5 | INH |
| 17 | HR-6 | INH |
| 18 | HR-7 | INH |
| 19 | HR-8 | INH |
| 20 | HR-9 | INH |
| 21 | HR-10 | INH |
| 22 | HR-11 | INH |
| 23 | HR-12 | INH |
| 24 | HR-13 | INH |
| 25 | HR-14 | INH |
| 26 | HR-15 | INH |
| 27 | HR-16 | INH |
| 28 | HR-17 | INH |
| 29 | HR-18 | INH |
| 30 | MDR-1 | RIF, INH, SM, KMA, OFLX |
| 31 | MDR-2 | RIF, INH, EMB, KMA, OFLX |
| 32 | MDR-3 | RIF, INH, SM, EMB, KMA |
| 33 | MDR-4 | RIF, INH, EMB, KMA |
| 34 | MDR-5 | RIF, INH |
| 35 | MDR-6 | RIF, INH |
| 36 | MDR-7 | RIF, INH, EMB, KMA |
| 37 | MDR-8 | RIF, INH |
| 38 | MDR-9 | RIF, INH, EMB, KMA, OFLX |
| 39 | MDR-10 | RIF, INH |
| 40 | MDR-11 | RIF, INH |
| 41 | MDR-12 | INH, RIF, SM |
| 42 | MDR-13 | INH, RIF, SM |
| 43 | MDR-14 | RIF, INH |
| 44 | MDR-15 | INH, RIF, SM, KMA |
| 45 | MDR-16 | INH, RIF, SM, KMA |
| 46 | MDR-17 | INH, RIF, SM |
| 47 | MDR-18 | INH, RIF, SM |

**Table S2 Primer sequences for amplification of mutation-associated genes, resistance-related genes and efflux pump genes**

|  | Genes |  | Primer sequences (5' →3') | Products size (bp) | Family |
| --- | --- | --- | --- | --- | --- |
| **mutation-associated genes** | *rpoB* | Forward | CGAATATCTGGTCCGCTTG | 571 | - |
|  |  | Reverse | GGTCAGGTACACGATCTC |  |  |
|  | *KatG* | Forward | CGGCGATGAGCGTTACAG | 458 | - |
|  |  | Reverse | CGTCCTTGGCGGTGTATTG |  |  |
|  | *inhA* | Forward | TGCCCAGAAAGGGATCCGTCATG | 455 | - |
|  |  | Reverse | ATGAGGAATGCGTCCGCGGA |  |  |
|  | *oxyR-ahpC* | Forward | GAGACCGGCTTCCGACCACC | 293 | - |
|  |  | Reverse | GCTGGTAGGCGGGGAATTGAT |  |  |
| **resistance-related genes** | *sigA* | Forward | GTGGCAGCGACCAAAGCAAG | 200 | - |
|  |  | Reverse | GTGTCCTGGGGTGCCGAG |  |  |
|  | 16S rRNA | Forward | CAAGGCTAAAACTCAAAGGA | 197 | - |
|  |  | Reverse | GGACTTAACCCAACATCTCA |  |  |
|  | *polA* | Forward | TTTCACTGCTCGATGACGAC | 186 | - |
|  |  | Reverse | TACCGGCACTTTCCATCTTC |  |  |
|  | *secA* | Forward | CACTACGAGGTCGATCTA | 180 | - |
|  |  | Reverse | GACGATGTAGTCCTTGTC |  |  |
|  | *Hsp65* | Forward | TCGAGACCAAGGAGCAGATT | 200 | - |
|  |  | Reverse | CACGAAGTACCCCGAGATGT |  |  |
|  | GAPDH | Forward | CAGGCTCAAGGGCATTCTGA | 183 | - |
|  |  | Reverse | AGCGTGACCAGATCAACCAG |  |  |
|  | *sigB* | Forward | TCTATCTGAACGGCATCGGC | 113 | - |
|  |  | Reverse | CTTCCGGGTTTCCAGCAGAT |  |  |
|  | *rpoB* | Forward | CCTGGAAGAGGTGCTCTACG | 219 | - |
|  |  | Reverse | GGGAAGTCACCCATGAACAC |  |  |
| **efflux pump genes** | Rv3065 | Forward | ATACCTCTTGTGCGCGATCT | 109 | SMR |
|  |  | Reverse | GCCATAACCCACTAGACAGC |  |  |
|  | Rv2836c | Forward | CCAATTGCCTTTTGCGGGAA | 127 | MATE |
|  |  | Reverse | CAACCAGACAAGCGGCAAAA |  |  |
|  | efpA | Forward | ATGGTAATGCCTGACATCC | 131 | MFS |
|  |  | Reverse | CTACGGGAAACCAACAAAG |  |  |
|  | Rv1410c | Forward | TGTGGTTCCTTATCGCCCTA | 126 | MFS |
|  |  | Reverse | GGGAAATAAGCCAGTAACCGT |  |  |
|  | Rv1250 | Forward | GCAGCCTTGGATTTGGGCGGTGAT | 131 | MFS |
|  |  | Reverse | GGACAAGCTGAAGTTCCGGTCGTT |  |  |
|  | Rv0876c | Forward | GGACCGATGAGTGGAGCGATCA | 133 | MFS |
|  |  | Reverse | ACTCGGCAATGGCGGTAGCA |  |  |
|  | Rv1819c | Forward | GCGTCGTAGTTGTTGCGGAAG | 165 | ABC |
|  |  | Reverse | TGGATGGAATCTGTCGGTGAGC |  |  |
|  | Rv0933 | Forward | CTGGACCCGACTACCACCGAGAA | 94 | ABC |
|  |  | Reverse | GCCTGGGCAAGGTTATGGGTC |  |  |
|  | Rv1217c | Forward | CGGTGAGGTTGGCGTAG | 150 | ABC |
|  |  | Reverse | CGGTCGGAATCTGGAAA |  |  |
|  | Rv1218c | Forward | CCGCAAGGCGTCTAGTGAA | 173 | ABC |
|  |  | Reverse | TGGACCCGTTGATGGAAAA |  |  |

**Table S3 PCR amplification conditions for amplification of resistance-related genes**

| ***rpoB* and *katG*** | | | ***inhA* and *oxyR-ahpC*** | | |
| --- | --- | --- | --- | --- | --- |
| **Temperature (℃)** | **Time** | **Cycles** | **Temperature (℃)** | **Time** | **Cycles** |
| 94 | 3min | 1 | 94 | 3min | 1 |
| 94 | 30s | 35 | 94 | 30s | 35 |
| 50 | 30s |  | 60 | 30s |  |
| 72 | 35s |  | 72 | 35s |  |
| 72 | 10min | 1 | 72 | 10min | 1 |

**Table S4 MIRU-VNTR typing and mutations of the isolates**

| **sample ID** | **MIRU-VNTR** | **mutations** |
| --- | --- | --- |
| H37Rv | 233226133321 | wt |
| DS-1 | 235125113323 | wt |
| DS-2 | 233325173633 | wt |
| DS-3 | 233325173633 | wt |
| DS-4 | 233325173633 | wt |
| DS-5 | 233325173633 | wt |
| RR-1 | 233325173623 | *rpoB*（526 CAC→GAC） |
| RR-2 | 233325173634 | *rpoB*（531 TCG→TTG） |
| RR-3 | 233325173633 | *rpoB*（526 CAC→TAC） |
| RR-4 | 233325163634 | *rpoB*（531 TCG→TTG） |
| RR-5 | 233325173623 | wt |
| HR-1 | 242225151322 | wt |
| HR-2 | 234126113322 | *katG*（315 AGC→ACC） |
| HR-3 | 232323153223 | *katG*（315 AGC→ACC） |
| HR-4 | 233325172433 | wt |
| HR-5 | 233525163522 | *katG*（315 AGC→ACC） |
| HR-6 | 232225152231 | *katG*（315 AGC→ACC） |
| HR-7 | 233325173633 | *katG*（315 AGC→ACC） |
| HR-8 | 232325152622 | *inhA*（-15 C→T） |
| HR-9 | 232325173323 | *katG*（315 AGC→ACC）、*inhA*（-15 C→T） |
| HR-10 | 233325173433 | *katG*（315 AGC→ACC） |
| HR-11 | 232325172322 | *katG*（315 AGC→ACC） |
| HR-12 | 252325162322 | *inhA*（-15 C→T） |
| HR-13 | 2333251103633 | *inhA*（-15 C→T） |
| HR-14 | 232325163323 | *inhA*（-15 C→T） |
| HR-15 | 213251611233 | wt |
| HR-16 | 213325163633 | wt |
| HR-17 | 233325173633 | *katG*（315 AGC→ACC） |
| HR-18 | 233325183631 | *katG*（315 AGC→ACC） |
| MDR-1 | 233325173533 | *rpoB*（516 TGG→TGT/517 ACC→TCC）*katG*（315 AGC→ACC） |
| MDR-2 | 232325173533 | *rpoB*（531 TCG→TTG）*katG*（315 AGC→ACC） |
| MDR-3 | 242125162322 | *rpoB*（526 CAC→CGA）*katG*（315 AGC→ACC） |
| MDR-4 | 233325183543 | *rpoB*（531 TCG→TTG）*katG*（315 AGC→ACC） |
| MDR-5 | 233325183533 | *rpoB*（531 TCG→TTG）*katG*（315 AGC→ACC） |
| MDR-6 | 252325153322 | *rpoB*（531 TCG→TTG）*katG*（315 AGC→ACC） |
| MDR-7 | 233325113533 | *rpoB*（531 TCG→TTG）*katG*（315 AGC→ACC） |
| MDR-8 | 233325173533 | *rpoB*（531 TCG→TTG）*katG*（315 AGC→ACC） |
| MDR-9 | 232325153421 | *rpoB*（531 TCG→TTG）*katG*（315 AGC→ACC） |
| MDR-10 | 233325103530 | *rpoB*（531 TCG→TTG）*katG*（315 AGC→ACC） |
| MDR-11 | 233325173543 | *rpoB*（511 CTG→CCG/512 AGC→GGC）*katG*（315 AGC→ACC） |
| MDR-12 | 2332251101523 | *rpoB*（531 TCG→TTG）*katG*（315 AGC→ACC） |
| MDR-13 | 233325173613 | *rpoB*（531 TCG→TTG）*katG*（315 AGC→ACC） |
| MDR-14 | 233325173633 | *rpoB*（509 AGC→CGC）*katG*（315 AGC→ACC） |
| MDR-15 | 233325173533 | *rpoB*（511 CTG→CCG/572 ATC→CTC）*katG*（315 AGC→ACC） |
| MDR-16 | 233225183033 | *rpoB*（511 CTG→CCG）*katG*（315 AGC→ACC） |
| MDR-17 | 233335294631 | *rpoB*（531 TCG→TTG）*katG*（315 AGC→ACC） |
| MDR-18 | 236126213220 | *rpoB*（517 ACC→TCC）*katG*（315 AGC→ACC） |
